# Dietary Factors and Cardiovascular Diseases: Comprehensive Insights from the National Health and Nutrition Examination Survey 2017–2020 and Mendelian Randomization Analysis

**DOI:** 10.3390/nu16223829

**Published:** 2024-11-08

**Authors:** Chaoqun Wang, Yikun Gao, Daniel Smerin, Mohammad Rohul Amin, Zhibiao Chen, Zhihong Jian, Lijuan Gu, Xiaoxing Xiong

**Affiliations:** 1 Department of Neurosurgery, Renmin Hospital of Wuhan University, Wuhan 430064, China; chaoqunwang2022@163.com (C.W.); rohulaminctgu@gmail.com (M.R.A.); zhihong@whu.edu.cn (Z.J.); 2 Central Laboratory, Renmin Hospital of Wuhan University, Wuhan 430064, China; 17339482028@163.com; 3 Department of Anesthesiology, Renmin Hospital of Wuhan University, Wuhan 430064, China; 4 UT Health San Antonio, Long School of Medicine, South Texas Research Facility, 8403 Floyd Curl Dr, San Antonio, TX 78229, USA; dsmerin@alumni.stanford.edu

**Keywords:** NHANES, mendelian randomization, dietary factors, cardiovascular diseases

## Abstract

Background: Cardiovascular diseases (CVDs) are a major public health concern. The impact of dietary components on CVD risk has been recognized, but their interactions require further investigation. This study aimed to examine the associations between major nutrient intake and CVD risk and to assess potential causal relationships via Mendelian randomization. Methods: We conducted a cross-sectional analysis using data from the National Health and Nutrition Examination Survey (NHANES) 2017–2020, with a sample size of 5464 adult participants. Nutrient intake was derived from two 24 h dietary recalls. Associations between four principal nutrients and CVD risk were evaluated via Mendelian randomization analysis. Additionally, weighted multivariable logistic regression analyses were performed to adjust for potential confounders, including age, sex, BMI, and other lifestyle factors. Results: An observational analysis revealed that increased log-transformed dietary fat intake was associated with reduced heart failure risk (OR = 0.722, 95% CI: 0.549–0.954). Log-transformed protein intake was protective against heart failure (OR = 0.645, 95% CI: 0.471–0.889), coronary artery disease (OR = 0.684, 95% CI: 0.504–0.931), and stroke (OR = 0.747, 95% CI: 0.568–0.988). IVW-MR analyses confirmed causal relationships between relative fat intake and heart failure risk (OR = 0.766, 95% CI: 0.598–0.982, *p* = 0.035) and between protein intake and stroke risk (OR = 0.993, 95% CI: 0.988–0.998, *p* = 0.010). MR analysis also revealed causal relationships between relative fat intake and coronary artery disease risk and between relative protein intake and hypertension risk. Conclusions: Both the observational and Mendelian randomization studies indicated that dietary fat is inversely associated with heart failure risk and that protein intake is correlated with reduced stroke risk. Future studies should investigate the optimal balance of macronutrients for CVD prevention, explore potential mechanisms underlying these associations, and consider long-term dietary interventions to validate these findings.

## 1. Introduction

Cardiovascular diseases (CVDs), including coronary artery disease (CAD), heart failure (HF), stroke, and hypertension, represent the foremost cause of mortality globally, accounting for approximately 17.9 million deaths annually, with a significant proportion attributed to modifiable risk factors such as lifestyle and dietary habits [1,2]. Emerging evidence from numerous studies highlights the critical role of dietary components in the etiology and progression of these conditions, emphasizing the necessity of delineating the complex interactions between diet and CVDs [3,4,5,6].

Macronutrients—carbohydrates, fats, proteins, and sugars—constitute essential elements of the human diet, influencing metabolic pathways and potentially modulating cardiovascular event risk. Despite extensive epidemiological research elucidating the effects of these nutrients on CVD risk, the results frequently appear inconsistent, which is attributable to confounders such as sample size, population heterogeneity, lifestyle factors, and genetic predispositions. For example, one study indicated that a low-carbohydrate diet (20% carbohydrates, 60% fats, and 20% proteins) could improve cardiometabolic risk profiles [7]. Conversely, in middle-aged Australian women, moderate carbohydrate intake (41.0% to 44.3% of total energy intake) was inversely associated with CVD risk but did not affect overall mortality [8]. However, a prospective cohort study of 110,497 participants from the UK Biobank revealed no association between total carbohydrate intake and CVD outcomes [4]. The role of dietary fats in CVDs has also been contentious. High-fat diets rich in choline are metabolized by the gut microbiota into trimethylamine (TMA), which promotes CVDs [9]. Additionally, a longitudinal study reviewing dietary fat consumption in China over 30 years (1990–2019) demonstrated a positive correlation between overall dietary fat intake and CVD incidence and mortality [10]. Conversely, other studies have reported no significant associations between total fat, saturated fat, or unsaturated fat and CVD risk [11]. The role of added sugars in cardiovascular health is widely debated. Many studies link high sugar intake, particularly from sugary beverages, to obesity, hypertension, type 2 diabetes, and CVD risk factors such as elevated triglycerides and insulin resistance [12,13,14]. The American Heart Association recommends limiting sugar intake to reduce these risks [15]. However, some researchers argue that the relationship is confounded by factors such as overall diet quality and lifestyle, and the differential impacts of sugars (e.g., fructose vs. glucose) remain contentious [16,17]. The impact of dietary protein on cardiovascular health is also complex. High intake of red and processed meats has been associated with increased CVD risk due to saturated fats and preservatives, whereas plant-based proteins have been linked to improved heart health [18,19,20]. However, recent studies suggest that lean red meat, in moderation, may not significantly increase CVD risk and that dairy proteins have mixed effects on blood pressure and lipid profiles [21,22,23].

Given these inconsistencies, a robust methodology is imperative to clarify the associations and causal effects between dietary nutrients and CVDs. Randomized controlled trials (RCTs), though considered the gold standard for establishing causal relationships, are often impractical because of their expense and complexity, particularly concerning dietary interventions and chronic diseases [24]. In addition, traditional observational studies often face limitations in establishing clear causal relationships because of confounding factors and potential reverse causation. For example, individuals may change their diet on the basis of health concerns, complicating the interpretation of data regarding the effects of sugar and protein intake. To address these challenges, Mendelian randomization (MR) offers a unique tool in this regard by utilizing genetic variants as instrumental variables to infer causal relationships between exposure factors and outcomes, effectively overcoming the confounding factors inherent in traditional observational studies and providing clearer insights into the potential causal relationships between exposures and outcomes. Therefore, MR is widely recognized as a complementary method to RCTs [25,26]. Additionally, the National Health and Nutrition Examination Survey (NHANES), conducted by the National Center for Health Statistics (NCHS), provides high-quality, nationally representative data that are instrumental in examining the relationship between diet and cardiovascular health [27].

This study utilizes NHANES data from 2017 to 2020 and integrates two-sample MR methods to rigorously analyze the associations and potential causal relationships between major macronutrient intake (carbohydrates, fats, proteins, and sugars) and the risk of significant CVDs, including HF, CAD, stroke, and hypertension. By combining observational data with genetic insights, this research aims to refine our understanding of how specific nutrients may influence CVD risk, thereby informing targeted nutritional interventions to enhance cardiovascular health outcomes.

## 2. Methods

### 2.1. Overall Study Design

There were two parts to the current investigation. The first utilized NHANES data to examine the associations between the intake of macronutrients—including carbohydrates, fats, proteins, and sugars—and CVDs, including HF, CAD, stroke, and hypertension, adjusting for multiple potential confounders. The second component assessed the causal relationships between genetically predicted levels of macronutrient intake and CVD outcomes via MR analysis of summary statistics from genome-wide association studies (GWASs).

### 2.2. Observational Study

#### 2.2.1. Sample Population in the NHANES

**Figure 1:**
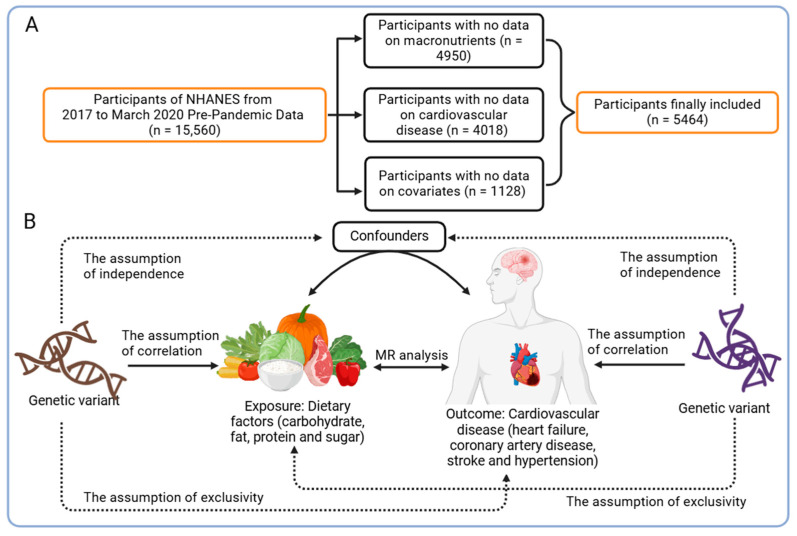
Observational analysis and Mendelian randomization form the basis of the overall study design: (**A**) flowchart of the observational study; (**B**) flowchart of the MR analysis, along with the three key assumptions for Mendelian randomization: Assumption 1: the exposure is closely associated with the genetic variant; Assumption 2: the genetic variant is not associated with confounding factors; Assumption 3: the genetic variant affects the outcome only through the exposure of interest. NHANES: National Health and Nutrition Survey, MR: Mendelian randomization. The images were adapted from biorender.com under the terms of the Non-Commercial Use License.

The NHANES 2017–March 2020 prepandemic dataset provided the data used in this investigation. Participants who were well informed about their intake of macronutrients and CVDs were included. After individuals with missing covariate data were excluded, a total of 5464 subjects were analyzed, as illustrated in Figure 1A. The NCHS Research Ethics Review Board provided ethical approval for NHANES involving human subjects, and all participants provided informed consent.

#### 2.2.2. Intake of Macronutrients

Macronutrient intake was assessed as an independent variable in the exposure analysis. Macronutrient data were acquired via the dietary interview component. Two 24 h dietary recall interviews were performed by all NHANES participants: one in person at a Mobile Examination Center (MEC) and one over the phone three to ten days later. Owing to the right-skewed distribution of macronutrient intake, the data were naturally log-transformed for normalization [28,29]. In summary, the natural logarithm of the average of the data from the two interviews was used.

#### 2.2.3. CVD Conditions

The National Center for Health Statistics (NCHS), a division of the Centers for Disease Control and Prevention (CDC), performed the study, which included laboratory testing, physical examinations, and household interviews [30]. The conditions of HF, CAD, stroke, and hypertension were determined through household interviews, which were based on a “yes” response to the following question: “Have you ever been told by a doctor that you have this condition?”

#### 2.2.4. Covariate Information

The covariates included age (years), sex (male or female), race, poverty–income ratio (PIR), education level, body mass index (BMI; kg/m^2^), smoking status, alcohol consumption, high cholesterol, and diabetes. Every covariate was regarded as a possible confounding factor in the association between the intake of macronutrients and CVDs. The participants were categorized into two age groups: <50 years and ≥50 years. The Mexican American, other Hispanic, non-Hispanic White, non-Hispanic Black, and other ethnic groups were among the groups included. Less than 9th grade, 9th to 11th grade (including 12th grade without a diploma), high school graduate or GED, some college or AA degree, and college graduate or higher were the five groups into which educational attainment was divided. PIR was divided into four groups: <1 (below the poverty line), 1–1.99, 2–3.99, and ≥4 (highest income group). Normal weight (less than 25 kg/m^2^), overweight (between 25 and 30 kg/m^2^), and obese (more than 30 kg/m^2^) were the three BMI categories. Smoking status was defined as “no” for individuals who had smoked fewer than 100 cigarettes in their lifetime and “yes” for those who had smoked 100 or more. Alcohol consumption was classified as “no” for individuals who had never consumed alcohol and “yes” for those who had consumed at least one alcoholic drink (excluding small tastes or sips). High cholesterol and diabetes were defined by a positive response to the following question: “Have you ever been told by a doctor that you have high cholesterol/diabetes?”

### 2.3. Statistical Analysis for Observational Study

Using NHANES sample weights and taking into consideration the intricacies of multistage cluster surveys, statistical analyses were carried out in accordance with CDC guidelines. Continuous data are expressed as the means with standard deviations, whereas categorical data are presented as percentages. For continuous variables, *t*-tests were applied, and associations between categorical variables were assessed via a weighted chi-square test. Multivariate logistic regression was employed to evaluate the independent association between macronutrient intake and CVD conditions across three models: Model 1 was unadjusted; Model 2 was adjusted for sex, age, and ethnicity; and Model 3 included adjustments for all relevant covariates. Penalized spline smooth curve fitting and weighted generalized additive models (GAMs) were used to explore nonlinear associations between macronutrient intake and CVD risk. Subgroup analyses were performed via stratified multivariate regression, with stratifications based on age, sex, ethnicity, education level, poverty–income ratio, BMI, smoking status, alcohol consumption, cholesterol level, and diabetes status. R software (version 4.3.0) was used for all the statistical analyses, and a significance threshold of 0.05 was chosen.

### 2.4. Mendelian Randomization

#### 2.4.1. Study Design

A two-sample MR was used to further validate the causal relationship between the intake of macronutrients and CVD conditions observed in the observational study based on the NHANES database. Causal inference primarily utilizes the inverse-variance weighted (IVW) MR method. By dividing the SNP-outcome association by the SNP–exposure association, this technique aggregates Wald ratio estimates for each SNP into a single causal estimate for each risk factor. The IVW results are objective if horizontal pleiotropy is absent. We conducted supplementary validation through Cochran’s Q test, MR-Egger regression, and leave-one-out sensitivity analysis to ensure the reliability of the results. An overview of the research design is presented in Figure 1B.

#### 2.4.2. Selection of Genetic Tools

Summary GWAS data for macronutrients, including carbohydrates, fat, protein, and sugar, were obtained from the Social Science Genetic Association Consortium (SSGAC). The sample sizes for GWASs were as follows: 235,391 participants for sugar intake research and 268,922 participants each for the fat, protein, and carbohydrate intake studies. Comprehensive dietary questionnaires covering either the subjects’ regular dietary habits or their diet from the previous day were used to measure the relative intake of carbohydrates, fat, protein, and sugar. The constant nutrient intake after adjusting for total calorie consumption was known as relative intake. Each GWAS dataset was subjected to defined quality control procedures by the study team to guarantee data accuracy and integrity [31]. The details of the macronutrient GWAS datasets used in our study are listed in Table 1.

Due to the limited availability of SNPs associated with macronutrient intake, we selected SNPs that were genome-wide significant (*p* < 5 × 10^−6^) [32]. Additionally, these SNPs showed paired linkage disequilibrium (LD), with a distance threshold of less than 10,000 kb and an r^2^ value of less than 0.01. Additionally, SNPs associated with possible confounders, such as diabetes, smoking, alcohol use, and BMI, were not included. These covariates had the following GWAS IDs: ebi-a-GCST006867 for diabetes, ukb-b-16878 for alcohol use, ebi-a-GCST90025994 for BMI, and ebi-a-GCST90029014 for smoking. The strength of the genetic instruments was assessed via the F statistic, which was calculated via the following formula: F = beta^2^/se^2^ [33]. Sufficient instrument strength was defined as an F statistic greater than 10. As instrumental variables (IVs), SNPs with an impact allele frequency >0.01 and F > 10 were chosen [34].

#### 2.4.3. Outcome Data

We acquired summary GWAS data for CVDs, including HF, CAD, stroke, and hypertension, from the IEU Open GWAS database (https://gwas.mrcieu.ac.uk/ accessed on 27 May 2024). All GWAS data were derived from populations of European ancestry, including HF involving 47,309 cases and 930,014 controls; CAD involving 352,063 individuals; stroke involving 6925 cases and 477,673 controls; and hypertension comprising 129,909 cases and 354,689 controls. The details of the GWAS datasets are described in Table 1.

### 2.5. Statistical Analysis for Mendelian Randomization

Maximum likelihood, MR-Egger regression, weighted median, and IVW were among the MR techniques used. Among these methods, the IVW method performed marginally better and was used as the main analytical method; the other three methods provided additional support for the findings.

Both MR-Egger and IVW were used to evaluate IV heterogeneity in conjunction with Cochran’s Q statistic; homogeneity was demonstrated by a Q-*p* value > 0.05. The MR-Egger approach, which employs weighted linear regression with an unconstrained intercept, was used to assess horizontal pleiotropy. Pleiotropy was shown by a significant departure of the intercept from zero (*p* value < 0.05). A leave-one-out sensitivity test, in which each IV was successively omitted to evaluate stability, was used to further investigate the robustness of the MR data.

The “TwoSampleMR”, “forestploter”, and “LDlinkR” packages were used in R (Version 4.3.0; R Foundation for Statistical Computing, Vienna, Austria) for all MR studies. A cutoff point of 0.05 was established for statistical significance. There was no need for ethical approval because the summary data used were publicly available.

## 3. Results

### 3.1. Population Characteristics of the NHANES

This study analyzed data from NHANES 2017–2020, which included 2606 men (47.694%) and 2858 women (52.306%). Table 2, Table 3, Table 4 and Table 5 present the baseline characteristics of the study population stratified by quartiles of intake for carbohydrates, fats, proteins, and sugars. Significant differences (*p* < 0.05) were observed across intake quartiles for age, sex, ethnicity, education level, poverty–income ratio, and smoking status. The participants in the highest quartiles of all four nutrients were generally older, more likely to be male, and predominantly non-Hispanic White. For example, in the carbohydrate quartiles, male representation increased from 34.407% in Q1 to 66.764% in Q4 (*p* < 0.001). Higher education and income levels were also associated with the highest quartile of nutrient intake (*p* < 0.001 for all nutrients). Body mass index (BMI) differences were statistically significant only across carbohydrate intake quartiles (*p* = 0.004). Diabetes incidence varied significantly across quartiles for carbohydrates (*p* < 0.001), fats (*p* = 0.014), and sugars (*p* < 0.001) but not for proteins (*p* = 0.093). A lower diabetes incidence was observed in the highest quartiles of carbohydrate and sugar intake. High cholesterol levels were significantly different across quartiles for carbohydrates (*p* = 0.001) and sugars (*p* = 0.034), with a lower prevalence in the highest intake quartiles. Cardiovascular disease indicators also varied across nutrient quartiles. HF incidence differed significantly for carbohydrates (*p* = 0.006) and proteins (*p* = 0.020), whereas coronary artery disease incidence varied only across carbohydrate quartiles (*p* = 0.044). Stroke incidence was significantly different across carbohydrate (*p* = 0.035) and protein (*p* < 0.001) quartiles. Hypertension incidence varied significantly across quartiles for all nutrients except sugars (*p* < 0.001 for carbohydrates, fats, and proteins; *p* = 0.122 for sugars).

These baseline characteristics highlight the complex associations between major dietary nutrients and cardiovascular disease risk factors, providing a foundation for further analysis of their potential impacts on cardiovascular health.

### 3.2. Associations Between Dietary Factors and CVD Incidence

Table 6 presents the associations between dietary nutrient intake and CVD outcomes, including HF, CAD, and stroke. The comprehensive results are available in Supplementary Material S1. The analysis employed three models: unadjusted (Model 1); adjusted for age, sex, and ethnicity (Model 2); and fully adjusted for all covariates (Model 3).

In the unadjusted model (Model 1), log-transformed carbohydrate (*p* < 0.01), fat (*p* < 0.01), and protein (*p* < 0.001) intakes were significantly inversely associated with HF. Protein intake was also inversely associated with CAD (*p* < 0.05) and stroke (*p* < 0.001). After adjusting for age, sex, and ethnicity (Model 2), these associations remained significant for fat intake and HF (*p* < 0.01), protein intake and HF (*p* < 0.01), protein intake and CAD (*p* < 0.05), and protein intake and stroke (*p* < 0.01). According to the fully adjusted model (Model 3), significant associations persisted between fat intake and HF (*p* < 0.05), as did protein intake and HF (*p* < 0.01), CAD (*p* < 0.05), and stroke (*p* < 0.05). The significant association between carbohydrate intake and HF risk observed in earlier models dissipated after full adjustment. Notably, the inverse association between protein intake and CVD outcomes demonstrated the most robustness across all the models. According to the fully adjusted model, protein intake was associated with lower odds of HF (OR: 0.645, 95% CI: 0.471–0.889), CAD (OR: 0.684, 95% CI: 0.504–0.931), and stroke (OR: 0.747, 95% CI: 0.568–0.988). Sugar intake was not significantly associated with any CVD outcome across the three models, with all *p* values and trend *p* values >0.05. These results indicate that higher intakes of fat and protein may be associated with lower risks of certain CVD outcomes, particularly HF. The consistent inverse relationship between protein intake and reduced risks of HF, CAD, and stroke across all the models suggests a potentially protective effect of increased protein consumption against these CVDs.

### 3.3. MR Analysis of Dietary Factors and CVDs

**Figure 2:**
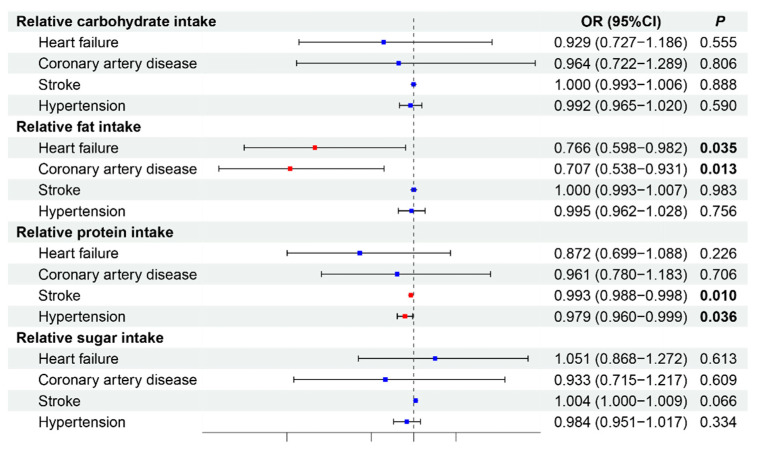
Causal effects of dietary factors on cardiovascular diseases by MR. Red *p* values indicate statistical significance (*p* < 0.05). The blue dots represent no statistically significant differences. OR, odds ratio.

Building on the observational study, we conducted MR analysis to investigate causal relationships between dietary nutrients and CVDs via data from GWAS databases. After removing confounding factors, we selected 46, 36, 39, and 41 SNPs for carbohydrates, fats, proteins, and sugars, respectively, as IVs to assess the causal relationships between these nutrients and CVDs (Supplementary Material S2). Given that IVW is the primary method used in MR analysis, we primarily present the IVW results [35]. The results of the MR analysis are presented in Figure 2, with sensitivity analyses shown in Table 7.

Our findings indicate that relative fat intake is protectively associated with HF (OR: 0.766, 95% CI: 0.598–0.982, *p* = 0.035) and CAD (OR: 0.707, 95% CI: 0.538–0.931, *p* = 0.013). Relative protein intake had a protective effect against stroke (OR: 0.993, 95% CI: 0.988–0.998, *p* = 0.010) and hypertension (OR: 0.979, 95% CI: 0.960–0.999, *p* = 0.036). Relative carbohydrate intake was not significantly associated with any CVD outcome (all *p* > 0.05). Similarly, relative sugar intake did not demonstrate significant causal relationships with the examined CVD outcomes, although there was a trend toward an increased risk of stroke (OR: 1.004 95% CI: 1.000–1.009, *p* = 0.066). Sensitivity analyses were conducted to assess the robustness of these findings (Table 7). The MR-Egger and IVW heterogeneity tests revealed varying degrees of heterogeneity across different nutrients and outcomes. Relative carbohydrate intake showed significant heterogeneity for all outcomes (*p* < 0.05). Relative fat intake demonstrated heterogeneity for stroke (*p* = 0.03) and hypertension (*p* < 0.001). Relative protein intake showed less heterogeneity, with no significant heterogeneity across outcomes (all *p* > 0.05), and relative sugar intake exhibited significant heterogeneity for stroke (*p* < 0.001). Despite the presence of heterogeneity in some analyses, the MR-Egger pleiotropy test revealed no evidence of horizontal pleiotropy for any of the nutrients across outcomes (all intercepts *p* > 0.05). This finding supports the validity of the MR results, suggesting that the observed associations are likely causal rather than due to pleiotropy.

These findings suggest that increased fat intake may causally reduce the risk of HF and CAD, whereas increased protein intake may causally lower the risk of stroke and hypertension. The absence of significant pleiotropy strengthens the reliability of these causal inferences, despite the presence of heterogeneity in some analyses.

### 3.4. Dose-Response Relationship Analysis

**Figure 3:**
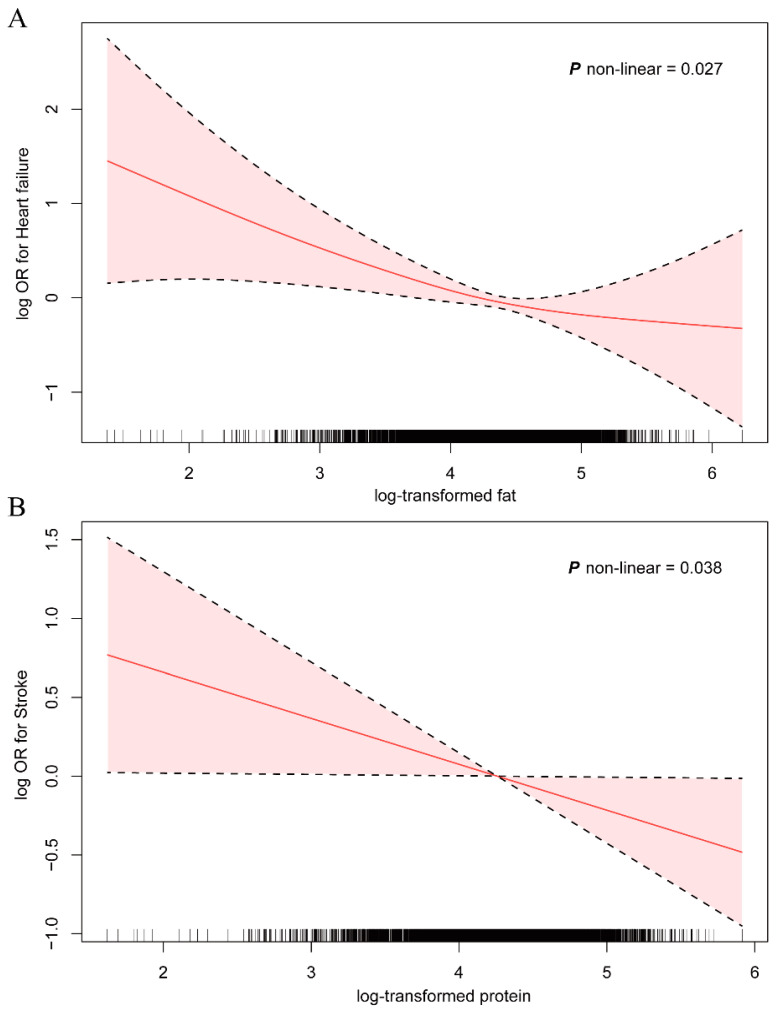
Associations of fat (**A**) and protein (**B**) intake with heart failure and stroke among NHANES 2017–2020 participants. ORs were adjusted for age, sex, ethnicity, education level, family income-poverty ratio, body mass index, smoking status, alcohol consumption, high cholesterol, and diabetes. The solid-line curves represent ORs, whereas the shaded areas denote 95% confidence intervals. Abbreviations: OR, odds ratio.

A generalized additive model (GAM) was employed to elucidate the dose-response relationships between fat intake and HF, as well as between protein intake and stroke, corroborating findings from both observational and MR studies. After adjusting for all covariates, nonlinear relationships emerged between log-transformed fat intake and heart failure risk, as well as between protein intake and stroke risk (*p* nonlinear < 0.05) (Figure 3).

As shown in Figure 3A, the log OR for HF initially decreased with increasing log-transformed fat intake and then plateaued with further increases. This pattern suggests a threshold effect whereby the protective benefit of fat intake stabilizes at higher intake levels. The nonlinear relationship was statistically significant (*p* nonlinear = 0.027), indicating that the association between fat intake and HF risk is not constant across all levels of intake. Similarly, Figure 3B illustrates the relationship between log-transformed protein intake and stroke risk. The log OR for stroke continuously decreased with increasing protein intake, indicating a progressively protective effect against stroke with increasing protein consumption. This nonlinear relationship was also statistically significant (*p* nonlinear = 0.038), suggesting that the protective effect of protein intake on stroke risk varies across different intake levels. In both cases, the shaded areas represent the 95% confidence intervals, which widen at the extremes of intake levels, indicating greater uncertainty in the estimates at very low and very high intake levels.

These dose-response analyses provide additional support for the findings from the observational and MR studies, demonstrating that the relationships between these dietary factors and cardiovascular outcomes are complex and nonlinear. The results suggest that moderate increases in fat and protein intake may have beneficial effects on HF and stroke risk, respectively, but these effects may not be uniform across all levels of intake.

### 3.5. Subgroup Analysis

**Figure 4:**
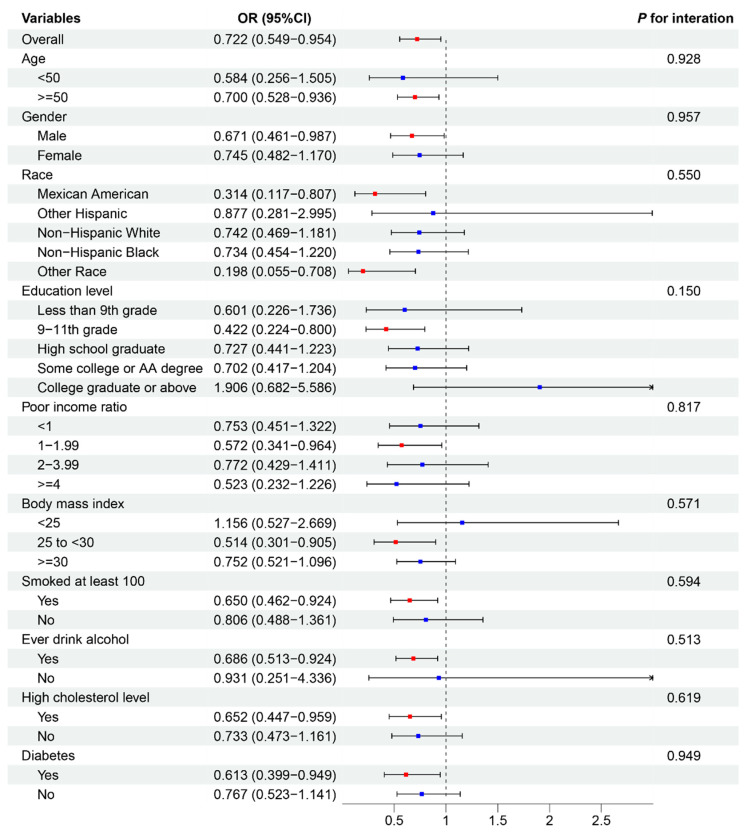
Subgroup analysis depicting the association between fat intake and heart failure. Red *p* values indicate statistical significance (*p* < 0.05). The blue dots represent no statistically significant differences. OR, odds ratio.

**Figure 5:**
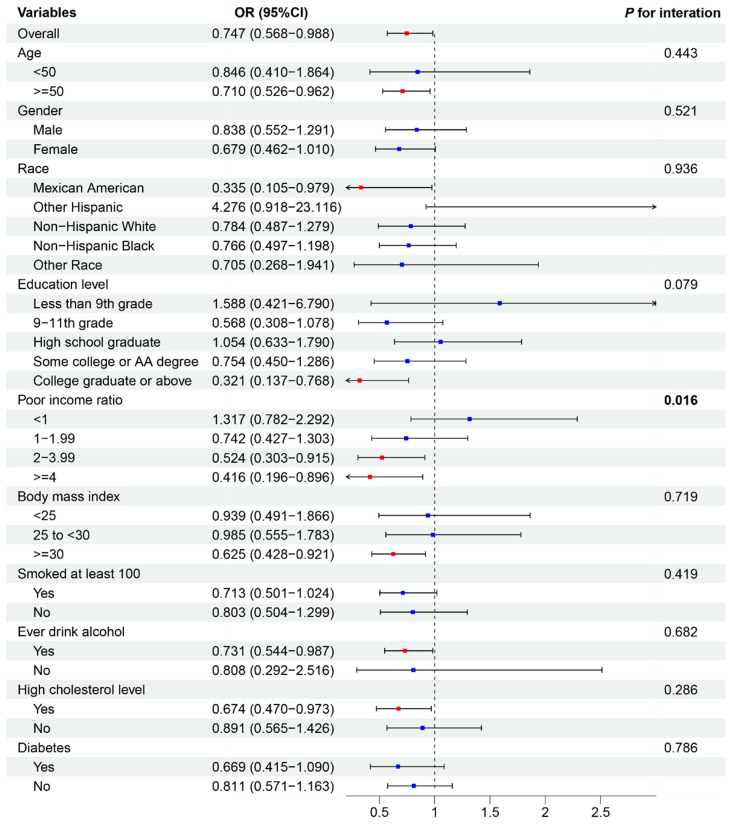
Subgroup analysis. This figure illustrates the association between protein intake and stroke. Red *p* values indicate statistical significance (*p* < 0.05). The blue dots represent no statistically significant differences. OR, odds ratio.

To assess the consistency of associations between dietary factors and CVD outcomes across various subgroups, we performed a subgroup analysis on the basis of the results of the observational study and MR analysis. Additionally, we evaluated interactions with age, sex, ethnicity, education level, poverty–income ratio, BMI, smoking status, alcohol consumption, and high cholesterol or diabetes status (Figure 4 and Figure 5).

Figure 4 depicts the association between fat intake and HF. The overall OR for this association was 0.727 (95% CI: 0.549–0.954), indicating a protective effect of fat intake against HF. This association remained relatively consistent across most subgroups, with no statistically significant interactions observed (all *p* values for interactions > 0.05). However, some variations in effect size were noted. The protective effect was stronger in males (OR: 0.671, 95% CI: 0.461–0.987) than in females (OR: 0.745, 95% CI: 0.482–1.170). Among ethnic groups, other ethnicities had the strongest protective effect (OR: 0.198, 95% CI: 0.055–0.708). Individuals with a medium poverty–income ratio (1–1.99) demonstrated a more pronounced protective effect (OR: 0.572, 95% CI: 0.341–0.964) than those with lower ratios. Figure 5 illustrates the association between protein intake and stroke incidence. The overall OR for this association was 0.747 (95% CI: 0.568–0.988), suggesting a protective effect of protein intake against stroke. This association was generally consistent across subgroups, with one notable exception. A significant interaction was observed for the poverty–income ratio (P for interaction = 0.016), indicating that the protective effect of protein intake on stroke risk may vary depending on income level.

Other subgroup analyses, including age, education level, BMI, smoking status, alcohol consumption, and the presence of high cholesterol or diabetes, did not reveal significant interactions for either the fat–HF or protein–stroke associations. These findings suggest that while the protective effects of fat intake against HF and protein intake against stroke are generally consistent across various demographic and health-related subgroups, there may be some variations in the strength of these associations, particularly with respect to sex, ethnicity, and income level.

## 4. Discussion

To our knowledge, this is the first comprehensive investigation delineating the linkages between four principal dietary nutrients and CVD risk, leveraging extensive observational and genetic datasets. Our analyses revealed that, within the framework of cross-sectional studies, multivariable-adjusted logistic regression models discerned robust associations between dietary fat intake and the risk of HF, as well as between protein consumption and stroke incidence. MR analyses support the conjecture of potential causal relationships, suggesting an inverse relationship between relative fat intake and HF, alongside a similar association between protein and stroke risk. Notably, both fat and protein intake were inversely correlated with these adverse cardiovascular outcomes. Additionally, our observations suggested possible linkages between protein intake and both HF and CAD; nevertheless, comprehensive MR analyses failed to corroborate specific causal interactions between protein intake and these cardiovascular conditions. Moreover, inferred genetically relative intakes of fat and protein were inversely correlated with CAD and hypertension, respectively, although such associations were absent in the observational component of the study.

Our observational dataset, adjusted for confounders, including age, sex, and race, consistently highlighted protective associations between high protein intake and HF, stroke, and CAD. These findings are congruent with other large-scale investigations suggesting that high-quality protein consumption confers cardiovascular benefits [3]. Nevertheless, some prior studies have reported an absence of significant associations between protein intake and cardiovascular health, suggesting that variability in protein effects is potentially modulated by other dietary components or genetic predispositions [36]. Additionally, recent investigations have illuminated the potential deleterious mechanisms by which excessive dietary protein and circulating leucine activate macrophage mammalian target of rapamycin (mTOR) signaling, thereby exacerbating atherosclerosis and increasing CVD risk at protein consumption thresholds of approximately 25 g per meal [6]. Our extensive study revealed an intriguing inverse correlation with HF. MR analyses further substantiated the protective role of fat intake against HF. Animal studies corroborate these findings, demonstrating that high-fat diets may ameliorate or even prevent HF through mechanisms involving the reversal of cardiac remodeling and dysfunction, thus suggesting the potential of high-fat diets as therapeutic interventions for HF [37]. In assessing the impact of carbohydrates on CVD risk, both observational studies and MR analyses, adjusted for covariates and confounding factors, revealed no significant associations or causal links between carbohydrate intake and CVD risk, in alignment with previous research findings [4]. The current literature indicates that carbohydrate quality, rather than quantity, significantly influences cardiovascular health [38,39]. The quality of carbohydrates, classified as simple or complex carbohydrates or high-glycemic index (GI) and low-GI foods, plays a crucial role in cardiovascular health [39]. High-quality carbohydrates, such as whole grains, legumes, and fruits, are associated with lower CVD risk [40,41,42,43]. Diets rich in high-GI carbohydrates have been associated with increased heart disease risk, whereas low-GI diets appear protective [44]. This may explain the absence of significant findings in our analyses, which did not discriminate between types of carbohydrates. Additionally, our results revealed no substantive associations between sugar intake and CVD outcomes in either the observational or MR analyses.

In contrast to conventional observational methodologies, MR mitigates confounding factors and strengthens the analysis of genetic predispositions that influence dietary behaviors and disease susceptibility. MR findings indicate that fat consumption is protective against HF and that protein intake mitigates stroke risk, corroborating our observational data and bolstering the validity of our conclusions. Dose-response assessments via GAMs have demonstrated significant nonlinear relationships between dietary fat and protein intake and cardiovascular outcomes, revealing a decrease in the protective effect of fat on HF risk beyond a specified threshold, with a parallel pattern observed for protein and stroke risk. Subgroup analyses further highlighted variations in the relationships between dietary fat and HF and between protein intake and stroke risk across demographic factors such as sex, ethnicity, and socioeconomic status. These disparities underscore the importance of considering demographic attributes in the formulation of nutritional guidelines. Notably, significant interactions in lower-income groups (interaction *p* = 0.016) suggest variable associations between protein intake and stroke risk across economic strata, potentially reflecting a confluence of genetic, environmental, or lifestyle factors that influence cardiovascular health. Such insights are pivotal for devising targeted nutritional interventions that accommodate cultural, economic, and biological diversity.

Nevertheless, MR analyses did not establish causal links between carbohydrate or protein intake and HF or between protein and CAD, thus differentiating these findings from observational data. Additionally, MR analysis indicated that each unit increase in protein intake reduces hypertension risk by 0.02%, which is supported by a study suggesting that higher protein intake is associated with a 26% reduction in hypertension risk per additional protein source [45]. Interestingly, the MR results suggest a protective association between fat intake and CAD. The cardiovascular effects of different types of fat are extremely complex because no effective method to distinguish dietary fat has been reported in the literature, so the conclusion that fat has a protective effect on CAD should be viewed with caution. Rigorous sensitivity analyses, as well as horizontal pleiotropy and heterogeneity tests, ensured the robustness of the MR results. In addition, discrepancies between observational and MR results may arise from unaccounted confounders in observational analyses [30], emphasizing the value of integrating MR methods to complement traditional epidemiological findings for more robust causal inferences.

This study’s significant strengths include the use of NHANES data, which offer a large, representative sample of the U.S. population, enhancing the generalizability of the findings. Additionally, the use of MR analysis mitigates the confounding factors and reverse causality inherent in traditional observational studies [46]. By using genetic variants as proxies for dietary intake, MR provides insights that are less likely to be biased by confounders or disease-related changes in diet. This study is the first to integrate NHANES data with MR techniques to explore macronutrient-CVD relationships, identifying new pathways for investigating dietary influences on disease. In both daily life and clinical settings, our findings have significant practical implications, bridging the gap between scientific research and real-world applications. The protective associations observed between fat intake and heart failure, as well as between protein intake and stroke, challenge conventional dietary wisdom and suggest a need for more nuanced nutritional guidance. In practice, these results could reshape dietary recommendations for cardiovascular health. Individuals and healthcare providers alike may consider a more balanced approach to macronutrient intake, moving away from broad restrictions on fat consumption and toward ensuring adequate intake of healthy fats and high-quality proteins. For example, patients at risk of heart failure may be advised to incorporate sources of healthy fats into their diets, whereas those with elevated stroke risk could focus on maintaining sufficient protein intake. Moreover, the nonlinear relationships observed in our dose-response analyses suggest that there may be optimal ranges for fat and protein intake, beyond which additional consumption may not confer extra benefits. This underscores the importance of balanced nutrition rather than extreme dietary approaches. However, these recommendations should be tailored to individual patient needs, and the quality of fat and protein sources should be considered. Our study offers several key lessons for both the scientific community and the general public. Researchers highlight the importance of integrating multiple methodologies, including observational studies and Mendelian randomization, to provide a more comprehensive understanding of diet-disease relationships. The discrepancies between our observational and MR results for some associations underscore the need for caution in interpreting epidemiological findings and the value of genetic approaches in nutrition research. For the broader scientific community, our work emphasizes the complexity of nutrition science and the need for more nuanced, personalized dietary recommendations. The observed variations across demographic subgroups suggest that one-size-fits-all dietary guidelines may be insufficient.

However, several limitations merit acknowledgment. One primary limitation is the lack of differentiation among types of macronutrients. Specifically, our analysis did not distinguish between different types of dietary fat (saturated, monounsaturated, and polyunsaturated fats), which have different effects on cardiovascular health and may also be one reason why we conclude that fat has a protective effect on CAD, contrary to most current studies. Research indicates that while saturated fats are not associated with cardiovascular risk, monounsaturated and polyunsaturated fats may have protective effects [5,47]. The distinction between animal and plant proteins was not addressed in our analysis. Increasing evidence suggests that the source of protein can differentially affect cardiovascular health outcomes. Specifically, animal proteins are associated with increased mortality risk, whereas plant proteins are linked to decreased cardiovascular risk [48,49,50]. By treating all protein sources equally, our analysis may obscure these critical differences. Similarly, this study did not differentiate between free sugars and nonfree sugars, which have substantially different health impacts. Higher consumption of free sugars (from sources such as high-sugar beverages, fruit juices, and sweets) is known to increase CVD risk [4]. Additionally, our study did not account for interactions between different nutrients, which could collectively influence cardiovascular health. These limitations highlight the complexity of dietary assessment in large epidemiological studies, where detailed dietary information is often sacrificed for broader data coverage. Thirdly, potential biases arising from population differences between NHANES (primarily comprising U.S. residents) and GWASs (primarily comprising Europeans) could affect the generalizability of our MR results owing to differences in genetic background and lifestyle factors. Finally, the fact that the NHANES data were based on self-report methods and did not contain specific biochemical measures is indeed a feature of the NHANES data and, at the same time, a limitation of our study. Future studies could incorporate specific biochemical measures such as blood glucose, HbA1c, cholesterol, and blood pressure levels for a more precise analysis. Building on our findings, we propose several key directions for future research. Studies should focus on differentiating between sources of diet to refine our understanding of their impacts on cardiovascular health. Long-term interventional trials are needed to confirm the efficacy of dietary modifications on the basis of our results. Additionally, investigating genetic interactions with dietary intake could pave the way for more personalized nutrition recommendations. Research into the biological mechanisms underlying the observed protective associations, particularly between fat intake and heart failure, may reveal new therapeutic targets. Future studies could focus on using GWAS datasets that better represent the NHANES population or conducting subgroup MR analyses within the NHANES dataset to investigate the impact of dietary factors on cardiovascular diseases in specific ethnic groups. Finally, developing and validating practical tools for implementing personalized dietary advice in clinical settings will be crucial for translating these findings into effective cardiovascular disease prevention strategies.

In conclusion, our study provides novel insights into the association between dietary nutrients and CVD risk, highlighting the pivotal role of dietary modifications in the prevention of CVDs at the population scale. Nonetheless, certain factors, including the potential underlying biological mechanisms, warrant further exploration. To surmount the limitations delineated in the present investigation, future research should endeavor to implement a meticulous categorization of nutrients and undertake expansive, multiethnic prospective studies to further the understanding and substantiation of these associations.

## 5. Conclusions

Our study, which integrates both observational data and MR analysis, demonstrated that increased dietary fat and protein intake are associated with a reduced risk of cardiovascular diseases, particularly heart failure and stroke. The protective effects of these macronutrients highlight the potential of targeted dietary modifications to improve cardiovascular health. Future research should focus on identifying the specific types of fats and proteins most beneficial for cardiovascular outcomes and further exploring the underlying mechanisms.

## Figures and Tables

**Table 1:** Descriptions of GWAS data used for analyses.

Trait	Year	Total No. or Case/Control	Ancestry	PMID	Dataset
Relative carbohydrate intake	2020	268,922	European	32393786	SSGAC19 consortium
Relative fat intake	2020	268,922	European	32393786	SSGAC19 consortium
Relative protein intake	2020	268,922	European	32393786	SSGAC19 consortium
Relative sugar intake	2020	235,391	European	32393786	SSGAC19 consortium
Heart failure	2020	47,309/930,014	European	31919418	ebi-a-GCST009541
Coronary artery disease	2021	352,063	European	34017140	ebi-a-GCST90013864
Stroke	2021	6925/477,673	European	33959723	ebi-a-GCST90038613
Hypertension	2021	129,909/354,689	European	33959723	ebi-a-GCST90038604

**Table 2:** Baseline characteristics of the participants according to quartiles of dietary carbohydrate intake.

Characteristic	Total (*n* = 5464)	Quartile 1 (*n* = 1366)	Quartile 2 (*n* = 1366)	Quartile 3 (*n* = 1366)	Quartile 4 (*n* = 1366)	*p*-Value
Age (year), mean value (SE)	50.477 (17.259)	52.197 (17.389)	51.203 (17.708)	51.114 (17.227)	47.396 (16.312)	<0.001
Gender (%)						<0.001
Male	2606 (47.694)	470 (34.407)	537 (39.312)	687 (50.293)	912 (66.764)	
Female	2858 (52.306)	896 (65.593)	829 (60.688)	679 (49.707)	454 (33.236)	
Race (%)						0.018
Mexican American	602 (11.018)	120 (8.785)	161 (11.786)	150 (10.981)	171 (12.518)	
Other Hispanic	510 (9.334)	134 (9.810)	136 (9.956)	114 (8.346)	126 (9.224)	
Non-Hispanic White	2094 (38.324)	512 (37.482)	527 (38.580)	525 (38.433)	530 (38.799)	
Non-Hispanic Black	1467 (26.848)	415 (30.381)	351 (25.695)	367 (26.867)	334 (24.451)	
Other Race—Including Multi-Racial	791 (14.477)	185 (13.543)	191 (13.982)	210 (15.373)	205 (15.007)	
Education level (%)						<0.001
Less than 9th grade	282 (5.161)	74 (5.417)	63 (4.612)	66 (4.832)	79 (5.783)	
9–11th grade (Includes 12th grade with no diploma)	552 (10.102)	159 (11.640)	119 (8.712)	120 (8.785)	154 (11.274)	
High school graduate/GED or equivalent	1281 (23.444)	356 (26.061)	309 (22.621)	316 (23.133)	300 (21.962)	
Some college or AA degree	1922 (35.176)	470 (34.407)	507 (37.116)	449 (32.870)	496 (36.310)	
College graduate or above	1427 (26.116)	307 (22.474)	368 (26.940)	415 (30.381)	337 (24.671)	
Poor income ratio (%)						0.001
<1	1022 (18.704)	284 (20.791)	226 (16.545)	231 (16.911)	281 (20.571)	
1–1.99	1393 (25.494)	356 (26.061)	345 (25.256)	341 (24.963)	351 (25.695)	
2–3.99	1525 (27.910)	343 (25.110)	389 (28.477)	389 (28.477)	404 (29.575)	
≥4	1524 (27.892)	383 (28.038)	406 (29.722)	405 (29.649)	330 (24.158)	
Body mass index (%)						0.004
<25	1380 (25.256)	318 (23.280)	331 (24.231)	360 (26.354)	371 (27.160)	
25 to <30	1668 (30.527)	403 (29.502)	395 (28.917)	427 (31.259)	443 (32.430)	
≥30	2416 (44.217)	645 (47.218)	640 (46.852)	579 (42.387)	552 (40.410)	
Smoked at least 100 (%)						<0.001
Yes	2321 (42.478)	590 (43.192)	524 (38.360)	567 (41.508)	640 (46.852)	
No	3143 (57.522)	776 (56.808)	842 (61.640)	799 (58.492)	726 (53.148)	
Ever drink alcohol (%)						0.090
Yes	5017 (91.819)	1250 (91.508)	1235 (90.410)	1265 (92.606)	1267 (92.753)	
No	447 (8.181)	116 (8.492)	131 (9.590)	101 (7.394)	99 (7.247)	
High cholesterol level (%)						0.001
Yes	1957 (35.816)	500 (36.603)	518 (37.921)	509 (37.262)	430 (31.479)	
No	3507 (64.184)	866 (63.397)	848 (62.079)	857 (62.738)	936 (68.521)	
Diabetes (%)						<0.001
Yes	846 (15.483)	259 (18.960)	233 (17.057)	197 (14.422)	157 (11.493)	
No	4618 (84.517)	1107 (81.040)	1133 (82.943)	1169 (85.578)	1209 (88.507)	
Heart failure (%)						0.006
Yes	192 (3.522)	57 (4.185)	61 (4.485)	43 (3.152)	31 (2.271)	
No	5259 (96.478)	1305 (95.815)	1299 (95.515)	1321 (96.848)	1334 (97.729)	
Coronary artery disease (%)						0.044
Yes	253 (4.640)	64 (4.692)	72 (5.294)	72 (5.275)	45 (3.302)	
No	5199 (95.360)	1300 (95.308)	1288 (94.706)	1293 (94.725)	1318 (96.698)	
Stroke (%)						0.035
Yes	269 (4.929)	85 (6.232)	60 (4.399)	70 (5.132)	54 (3.956)	
No	5188 (95.071)	1279 (93.768)	1304 (95.601)	1294 (94.868)	1311 (96.044)	
High blood pressure (%)						<0.001
Yes	2073 (37.939)	567 (41.508)	509 (37.262)	545 (39.898)	452 (33.089)	
No	3391 (62.061)	799 (58.492)	857 (62.738)	821 (60.102)	914 (66.911)	

**Table 3:** Baseline characteristics of the participants according to quartiles of dietary fat intake.

Characteristic	Total (*n* = 5464)	Quartile 1 (*n* = 1366)	Quartile 2 (*n* = 1366)	Quartile 3 (*n* = 1366)	Quartile 4 (*n* = 1366)	*p*-Value
Age (year), mean value (SE)	50.477 (17.259)	52.165 (17.406)	51.054 (17.481)	50.518 (17.175)	48.172 (16.738)	<0.001
Gender (%)						<0.001
Male	2606 (47.694)	474 (34.700)	527 (38.580)	691 (50.586)	914 (66.911)	
Female	2858 (52.306)	892 (65.300)	839 (61.420)	675 (49.414)	452 (33.089)	
Race (%)						<0.001
Mexican American	602 (11.018)	147 (10.761)	153 (11.201)	161 (11.786)	141 (10.322)	
Other Hispanic	510 (9.334)	165 (12.079)	126 (9.224)	118 (8.638)	101 (7.394)	
Non-Hispanic White	2094 (38.324)	404 (29.575)	545 (39.898)	557 (40.776)	588 (43.045)	
Non-Hispanic Black	1467 (26.848)	413 (30.234)	323 (23.646)	351 (25.695)	380 (27.818)	
Other Race—Including Multi-Racial	791 (14.477)	237 (17.350)	219 (16.032)	179 (13.104)	156 (11.420)	
Education level (%)						<0.001
Less than 9th grade	282 (5.161)	114 (8.346)	55 (4.026)	69 (5.051)	44 (3.221)	
9–11th grade (Includes 12th grade with no diploma)	552 (10.102)	168 (12.299)	125 (9.151)	127 (9.297)	132 (9.663)	
High school graduate/GED or equivalent	1281 (23.444)	337 (24.671)	318 (23.280)	320 (23.426)	306 (22.401)	
Some college or AA degree	1922 (35.176)	446 (32.650)	485 (35.505)	469 (34.334)	522 (38.214)	
College graduate or above	1427 (26.116)	301 (22.035)	383 (28.038)	381 (27.892)	362 (26.501)	
Poor income ratio (%)						<0.001
<1	1022 (18.704)	318 (23.280)	232 (16.984)	235 (17.204)	237 (17.350)	
1–1.99	1393 (25.494)	381 (27.892)	357 (26.135)	328 (24.012)	327 (23.939)	
2–3.99	1525 (27.910)	344 (25.183)	361 (26.428)	431 (31.552)	389 (28.477)	
≥4	1524 (27.892)	323 (23.646)	416 (30.454)	372 (27.233)	413 (30.234)	
Body mass index (%)						0.807
<25	1380 (25.256)	365 (26.720)	346 (25.329)	338 (24.744)	331 (24.231)	
25 to <30	1668 (30.527)	418 (30.600)	416 (30.454)	414 (30.307)	420 (30.747)	
≥30	2416 (44.217)	583 (42.679)	604 (44.217)	614 (44.949)	615 (45.022)	
Smoked at least 100 (%)						<0.001
Yes	2321 (42.478)	537 (39.312)	540 (39.531)	582 (42.606)	662 (48.463)	
No	3143 (57.522)	829 (60.688)	826 (60.469)	784 (57.394)	704 (51.537)	
Ever drink alcohol (%)						<0.001
Yes	5017 (91.819)	1179 (86.310)	1258 (92.094)	1276 (93.411)	1304 (95.461)	
No	447 (8.181)	187 (13.690)	108 (7.906)	90 (6.589)	62 (4.539)	
High cholesterol level (%)						0.853
Yes	1957 (35.816)	491 (35.944)	494 (36.164)	496 (36.310)	476 (34.846)	
No	3507 (64.184)	875 (64.056)	872 (63.836)	870 (63.690)	890 (65.154)	
Diabetes (%)						0.014
Yes	846 (15.483)	249 (18.228)	201 (14.714)	200 (14.641)	196 (14.348)	
No	4618 (84.517)	1117 (81.772)	1165 (85.286)	1166 (85.359)	1170 (85.652)	
Heart failure (%)						0.123
Yes	192 (3.522)	60 (4.412)	50 (3.663)	44 (3.233)	38 (2.784)	
No	5259 (96.478)	1300 (95.588)	1315 (96.337)	1317 (96.767)	1327 (97.216)	
Coronary artery disease (%)						0.784
Yes	253 (4.640)	65 (4.772)	60 (4.409)	69 (5.059)	59 (4.322)	
No	5199 (95.360)	1297 (95.228)	1301 (95.591)	1295 (94.941)	1306 (95.678)	
Stroke (%)						0.013
Yes	269 (4.929)	78 (5.710)	82 (6.021)	51 (3.742)	58 (4.246)	
No	5188 (95.071)	1288 (94.290)	1280 (93.979)	1312 (96.258)	1308 (95.754)	
High blood pressure (%)						0.059
Yes	2073 (37.939)	546 (39.971)	539 (39.458)	497 (36.384)	491 (35.944)	
No	3391 (62.061)	820 (60.029)	827 (60.542)	869 (63.616)	875 (64.056)	

**Table 4:** Baseline characteristics of the participants according to quartiles of dietary protein intake.

Characteristic	Total (*n* = 5464)	Quartile 1 (*n* = 1366)	Quartile 2 (*n* = 1366)	Quartile 3 (*n* = 1366)	Quartile 4 (*n* = 1366)	*p*-Value
Age (year), mean value (SE)	50.477 (17.259)	52.239 (17.445)	51.972 (17.812)	50.034 (17.168)	47.665 (16.200)	<0.001
Gender (%)						<0.001
Male	2606 (47.694)	399 (29.209)	504 (36.896)	722 (52.855)	981 (71.816)	
Female	2858 (52.306)	967 (70.791)	862 (63.104)	644 (47.145)	385 (28.184)	
Race (%)						<0.001
Mexican American	602 (11.018)	120 (8.785)	131 (9.590)	167 (12.225)	184 (13.470)	
Other Hispanic	510 (9.334)	134 (9.810)	124 (9.078)	112 (8.199)	140 (10.249)	
Non-Hispanic White	2094 (38.324)	475 (34.773)	542 (39.678)	544 (39.824)	533 (39.019)	
Non-Hispanic Black	1467 (26.848)	448 (32.796)	370 (27.086)	345 (25.256)	304 (22.255)	
Other Race—Including Multi-Racial	791 (14.477)	189 (13.836)	199 (14.568)	198 (14.495)	205 (15.007)	
Education level (%)						<0.001
Less than 9th grade	282 (5.161)	81 (5.930)	67 (4.905)	66 (4.832)	68 (4.978)	
9–11th grade (Includes 12th grade with no diploma)	552 (10.102)	166 (12.152)	118 (8.638)	136 (9.956)	132 (9.663)	
High school graduate/GED or equivalent	1281 (23.444)	362 (26.501)	333 (24.378)	289 (21.157)	297 (21.742)	
Some college or AA degree	1922 (35.176)	476 (34.846)	471 (34.480)	484 (35.432)	491 (35.944)	
College graduate or above	1427 (26.116)	281 (20.571)	377 (27.599)	391 (28.624)	378 (27.672)	
Poor income ratio (%)						<0.001
<1	1022 (18.704)	303 (22.182)	244 (17.862)	247 (18.082)	228 (16.691)	
1–1.99	1393 (25.494)	393 (28.770)	358 (26.208)	325 (23.792)	317 (23.206)	
2–3.99	1525 (27.910)	368 (26.940)	389 (28.477)	370 (27.086)	398 (29.136)	
≥4	1524 (27.892)	302 (22.108)	375 (27.452)	424 (31.040)	423 (30.966)	
Body mass index (%)						0.245
<25	1380 (25.256)	327 (23.939)	379 (27.745)	337 (24.671)	337 (24.671)	
25 to <30	1668 (30.527)	411 (30.088)	414 (30.307)	414 (30.307)	429 (31.406)	
≥30	2416 (44.217)	628 (45.974)	573 (41.947)	615 (45.022)	600 (43.924)	
Smoked at least 100 (%)						0.030
Yes	2321 (42.478)	556 (40.703)	552 (40.410)	596 (43.631)	617 (45.168)	
No	3143 (57.522)	810 (59.297)	814 (59.590)	770 (56.369)	749 (54.832)	
Ever drink alcohol (%)						<0.001
Yes	5017 (91.819)	1200 (87.848)	1245 (91.142)	1270 (92.972)	1302 (95.315)	
No	447 (8.181)	166 (12.152)	121 (8.858)	96 (7.028)	64 (4.685)	
High cholesterol level (%)						0.541
Yes	1957 (35.816)	495 (36.237)	503 (36.823)	491 (35.944)	468 (34.261)	
No	3507 (64.184)	871 (63.763)	863 (63.177)	875 (64.056)	898 (65.739)	
Diabetes (%)						0.093
Yes	846 (15.483)	229 (16.764)	209 (15.300)	223 (16.325)	185 (13.543)	
No	4618 (84.517)	1137 (83.236)	1157 (84.700)	1143 (83.675)	1181 (86.457)	
Heart failure (%)						0.020
Yes	192 (3.522)	61 (4.482)	56 (4.103)	36 (2.643)	39 (2.861)	
No	5259 (96.478)	1300 (95.518)	1309 (95.897)	1326 (97.357)	1324 (97.139)	
Coronary artery disease (%)						0.562
Yes	253 (4.640)	64 (4.702)	72 (5.282)	59 (4.322)	58 (4.255)	
No	5199 (95.360)	1297 (95.298)	1291 (94.718)	1306 (95.678)	1305 (95.745)	
Stroke (%)						<0.001
Yes	269 (4.929)	94 (6.902)	70 (5.136)	56 (4.100)	49 (3.587)	
No	5188 (95.071)	1268 (93.098)	1293 (94.864)	1310 (95.900)	1317 (96.413)	
High blood pressure (%)						0.006
Yes	2073 (37.939)	559 (40.922)	539 (39.458)	486 (35.578)	489 (35.798)	
No	3391 (62.061)	807 (59.078)	827 (60.542)	880 (64.422)	877 (64.202)	

**Table 5:** Baseline characteristics of the participants according to quartiles of dietary sugar intake.

Characteristic	Total (*n* = 5464)	Quartile 1 (*n* = 1366)	Quartile 2 (*n* = 1366)	Quartile 3 (*n* = 1366)	Quartile 4 (*n* = 1366)	*p*-Value
Age (year), mean value (SE)	50.477 (17.259)	50.822 (17.317)	51.272 (17.509)	50.810 (17.523)	49.005 (16.604)	0.003
Gender (%)						<0.001
Male	2606 (47.694)	556 (40.703)	582 (42.606)	652 (47.731)	816 (59.736)	
Female	2858 (52.306)	810 (59.297)	784 (57.394)	714 (52.269)	550 (40.264)	
Race (%)						<0.001
Mexican American	602 (11.018)	149 (10.908)	162 (11.859)	149 (10.908)	142 (10.395)	
Other Hispanic	510 (9.334)	145 (10.615)	132 (9.663)	123 (9.004)	110 (8.053)	
Non-Hispanic White	2094 (38.324)	506 (37.042)	492 (36.018)	515 (37.701)	581 (42.533)	
Non-Hispanic Black	1467 (26.848)	345 (25.256)	349 (25.549)	395 (28.917)	378 (27.672)	
Other Race—Including Multi-Racial	791 (14.477)	221 (16.179)	231 (16.911)	184 (13.470)	155 (11.347)	
Education level (%)						<0.001
Less than 9th grade	282 (5.161)	89 (6.515)	64 (4.685)	60 (4.392)	69 (5.051)	
9–11th grade (Includes 12th grade with no diploma)	552 (10.102)	147 (10.761)	133 (9.736)	121 (8.858)	151 (11.054)	
High school graduate/GED or equivalent	1281 (23.444)	327 (23.939)	307 (22.474)	302 (22.108)	345 (25.256)	
Some college or AA degree	1922 (35.176)	476 (34.846)	454 (33.236)	488 (35.725)	504 (36.896)	
College graduate or above	1427 (26.116)	327 (23.939)	408 (29.868)	395 (28.917)	297 (21.742)	
Poor income ratio (%)						<0.001
<1	1022 (18.704)	269 (19.693)	228 (16.691)	226 (16.545)	299 (21.889)	
1–1.99	1393 (25.494)	345 (25.256)	347 (25.403)	333 (24.378)	368 (26.940)	
2–3.99	1525 (27.910)	342 (25.037)	375 (27.452)	401 (29.356)	407 (29.795)	
≥4	1524 (27.892)	410 (30.015)	416 (30.454)	406 (29.722)	292 (21.376)	
Body mass index (%)						0.662
<25	1380 (25.256)	344 (25.183)	329 (24.085)	359 (26.281)	348 (25.476)	
25 to <30	1668 (30.527)	401 (29.356)	428 (31.332)	407 (29.795)	432 (31.625)	
≥30	2416 (44.217)	621 (45.461)	609 (44.583)	600 (43.924)	586 (42.899)	
Smoked at least 100 (%)						<0.001
Yes	2321 (42.478)	572 (41.874)	546 (39.971)	540 (39.531)	663 (48.536)	
No	3143 (57.522)	794 (58.126)	820 (60.029)	826 (60.469)	703 (51.464)	
Ever drink alcohol (%)						0.077
Yes	5017 (91.819)	1239 (90.703)	1245 (91.142)	1260 (92.240)	1273 (93.192)	
No	447 (8.181)	127 (9.297)	121 (8.858)	106 (7.760)	93 (6.808)	
High cholesterol level (%)						0.034
Yes	1957 (35.816)	484 (35.432)	523 (38.287)	499 (36.530)	451 (33.016)	
No	3507 (64.184)	882 (64.568)	843 (61.713)	867 (63.470)	915 (66.984)	
Diabetes (%)						<0.001
Yes	846 (15.483)	270 (19.766)	242 (17.716)	191 (13.982)	143 (10.469)	
No	4618 (84.517)	1096 (80.234)	1124 (82.284)	1175 (86.018)	1223 (89.531)	
Heart failure (%)						0.359
Yes	192 (3.522)	55 (4.035)	53 (3.894)	41 (3.010)	43 (3.150)	
No	5259 (96.478)	1308 (95.965)	1308 (96.106)	1321 (96.990)	1322 (96.850)	
Coronary artery disease (%)						0.736
Yes	253 (4.640)	61 (4.479)	70 (5.128)	64 (4.696)	58 (4.258)	
No	5199 (95.360)	1301 (95.521)	1295 (94.872)	1299 (95.304)	1304 (95.742)	
Stroke (%)						0.748
Yes	269 (4.929)	69 (5.059)	66 (4.835)	73 (5.356)	61 (4.469)	
No	5188 (95.071)	1295 (94.941)	1299 (95.165)	1290 (94.644)	1304 (95.531)	
High blood pressure (%)						0.122
Yes	2073 (37.939)	533 (39.019)	528 (38.653)	531 (38.873)	481 (35.212)	
No	3391 (62.061)	833 (60.981)	838 (61.347)	835 (61.127)	885 (64.788)	

**Table 6:** The results of multivariable-adjusted logistic regression.

	Characteristic	Log-Transformed	Q1	Q2	Q3	Q4	*p*-Trend
Carbohydrate	Heart failure						
	Model 1	0.645 (0.494–0.849) **	1.00	1.103 (0.774–1.577)	0.729 (0.490–1.077)	0.513 (0.329–0.786) **	<0.001
	Model 2	0.694 (0.517–0.941) *	1.00	1.122 (0.779–1.619)	0.710 (0.471–1.061)	0.611 (0.384–0.956) *	0.008
	Model 3	0.791 (0.585–1.079)	1.00	1.269 (0.871–1.854)	0.845 (0.556–1.279)	0.714 (0.444–1.131)	0.076
Fat	Heart failure						
	Model 1	0.677 (0.530–0.872) **	1.00	0.764 (0.524–1.108)	0.733 (0.500–1.067)	0.579 (0.384–0.862) **	0.008
	Model 2	0.691 (0.530–0.91) **	1.00	0.778 (0.528–1.139)	0.748 (0.504–1.103)	0.649 (0.422–0.987) *	0.044
	Model 3	0.722 (0.549–0.954) *	1.00	0.794 (0.533–1.177)	0.786 (0.522–1.177)	0.677 (0.433–1.046)	0.088
Protein	Heart failure						
	Model 1	0.574 (0.437–0.760) ***	1.00	0.858 (0.595–1.232)	0.594 (0.395–0.882) *	0.625 (0.418–0.923) *	0.005
	Model 2	0.593 (0.435–0.815) **	1.00	0.818 (0.562–1.187)	0.593 (0.388–0.897) *	0.730 (0.474–1.113)	0.051
	Model 3	0.645 (0.471–0.889) **	1.00	0.896 (0.610–1.315)	0.622 (0.402–0.952) *	0.781 (0.502–1.204)	0.101
	Coronary artery disease						
	Model 1	0.751 (0.582–0.974) *	1.00	1.092 (0.780–1.530)	0.864 (0.605–1.230)	0.879 (0.616–1.250)	0.272
	Model 2	0.680 (0.506–0.918) *	1.00	0.997 (0.700–1.422)	0.738 (0.504–1.077)	0.842 (0.571–1.239)	0.188
	Model 3	0.684 (0.504–0.931) *	1.00	0.989 (0.687–1.424)	0.664 (0.446–0.983) *	0.778 (0.519–1.162)	0.077
	Stroke						
	Model 1	0.566 (0.445–0.724) ***	1.00	0.73 (0.533–0.997) *	0.553 (0.393–0.772) ***	0.481 (0.336–0.680) ***	<0.001
	Model 2	0.649 (0.495–0.857) **	1.00	0.719 (0.520–0.989) *	0.605 (0.423–0.856) **	0.622 (0.424–0.903) *	0.004
	Model 3	0.747 (0.568–0.988) *	1.00	0.769 (0.553–1.065)	0.650 (0.452–0.928) *	0.680 (0.460–0.996)	0.020

Data are presented as OR (95%CI). * *p* < 0.05, ** *p* < 0.01, *** *p* < 0.001. Model 1: no covariates were adjusted. Model 2: adjusted for age, sex, and race. Model 3: further adjusted for all covariates.

**Table 7:** Heterogeneity and pleiotropy tests.

Exposure	Outcome	Heterogeneity Test MR–Egger		Heterogeneity Test IVW		Pleiotropy Test MR–Egger	
		Q	*p*	Q	*p*	Intercept	*p*
Relative carbohydrate intake	Heart failure	70.511	<0.001	78.254	<0.001	−0.013	0.051
	Coronary artery disease	82.673	<0.001	82.675	<0.001	<0.001	0.976
	Stroke	64.774	0.010	64.791	0.014	<0.001	0.918
	Hypertension	100.365	<0.001	101.891	<0.001	0.001	0.440
Relative fat intake	Heart failure	34.434	0.077	35.089	0.087	0.005	0.506
	Coronary artery disease	33.992	0.085	35.359	0.082	0.008	0.336
	Stroke	44.966	0.030	45.120	0.038	<0.001	0.754
	Hypertension	61.165	<0.001	61.349	<0.001	<0.001	0.782
Relative protein intake	Heart failure	40.098	0.065	40.298	0.079	0.002	0.711
	Coronary artery disease	25.615	0.646	27.551	0.594	0.007	0.175
	Stroke	32.983	0.517	32.986	0.566	<0.001	0.957
	Hypertension	27.338	0.605	29.861	0.524	−0.001	0.123
Relative sugar intake	Heart failure	31.477	0.392	34.639	0.298	−0.011	0.093
	Coronary artery disease	57.634	0.003	57.991	0.003	−0.004	0.665
	Stroke	131.978	<0.001	132.299	<0.001	<0.001	0.776
	Hypertension	29.916	0.789	29.996	0.820	<0.001	0.778

## Data Availability

All the data analyzed in this study are available from the corresponding author upon reasonable request. The data are not publicly available due to the policy of SSGAC.

## Supplementary Materials

The following supporting information can be downloaded at: https://www.mdpi.com/article/10.3390/nu16223829/s1, S1: Association between dietary factors and cardiovascular diseases; S2: SNPs.
